# Transcriptome analysis reveals a complex interplay between resistance and effector genes during the compatible lentil-*Colletotrichum lentis* interaction

**DOI:** 10.1038/srep42338

**Published:** 2017-02-10

**Authors:** Vijai Bhadauria, Perumal Vijayan, Yangdou Wei, Sabine Banniza

**Affiliations:** 1 Crop Development Centre/Department of Plant Sciences, University of Saskatchewan, Saskatoon, Canada; 2 Department of Biology, University of Saskatchewan, Saskatoon, Canada

## Abstract

*Colletotrichum lentis* is a hemibiotrophic pathogen and causes anthracnose on lentil. To understand the molecular mechanism underlying the symptomatic phase of infection, a cDNA plasmid library was developed from the susceptible lentil cultivar Eston infected with an isolate of the virulent race 0 of *C. lentis*. The library was sequenced on the Sanger sequencing platform, generating a total of 11,094 expressed sequence tags (ESTs) representing 3,488 unigenes. Mapping of unigenes onto the *C. lentis* and the *L. culinaris* genomes resulted in the identification of 2,418 unigenes of fungal origin and 1,070 unigenes of plant origin. Gene ontology term analysis of unigenes revealed that the transcriptome contained 22 candidate effectors, such as *in planta* induced *ToxB* and *CyanoVirin-N*, and 26 resistance genes, including suppressor of npr1-1 constitutive 1 and dirigent. Comparative genomics analyses revealed that three of the candidate effectors are likely located in the subtelomeric regions, and two of them show no synteny with the closely related species *C. higginsianum*, suggesting genomic rearrangements, such as translocation during speciation to colonize different niches. The data suggest a complex molecular interplay between disease resistance proteins and effectors during compatible interaction in which the pathogen exploits defense responses mounted by the host.

Lentil (*Lens culinaris* Medik.), an important source of dietary protein and fiber, ranks fifth among pulse crops worldwide in terms of production, and Canada is the leading exporter of lentils in the world^1^. Anthracnose caused by *Colletotrichum lentis* Damm (previously misidentified as *C. truncatum* (Schwein.) Andrus & W.D. Moore) has become the economically most important disease of Canadian lentil accounting for up to 70% crop loss under high disease pressure^2^,^3^. The pathogen employs a hemibiotrophic infection strategy to colonize lentil plants, which includes an initial symptomless biotrophic phase characterized by thick biotrophic primary hyphae followed by a symptomatic phase characterized by thin necrotrophic secondary hyphae.

Inducible innate immunity in plants is bi-layered and has evolved to prevent microbes from becoming pathogens. The first layer that acts at the cell surface level recognizes pathogen signatures conserved across microbes and is known as microbe-associated molecular patterns-triggered immunity (MTI). However, adapted pathogens overcome MTI by secreting intracellular effectors that block detection signals by inactivating MAPK. In response, plants recruit intracellular resistance (R) proteins to detect effectors for downstream defense response elicitation. Most of these proteins contain NBS and LRR domains^4^.

Genomic plasticity due to the co-evolutionary plant-pathogen arms race has triggered an uneven rate of evolution across pathogen genomes, which has led to compartmentalization of *in planta* induced genes, especially effectors. These specialized genes are an excellent example of the extended phenotype^5^ as they exert their activity in hosts by evading the NBS-LRR R protein receptor-mediated plant detection. These compartments enriched in virulence effector genes are located in gene-sparse subtelomeric regions or on conditionally dispensable chromosomes, which are gained through horizontal transfer from other fungal species^6^. High genetic variability in these compartments led to the emergence of new pathogen lineages capable of broadening host ranges by jumping hosts or enhancing virulence on existing hosts.

Two pathogenic races were described in the population of *C. lentis* isolated from lentil grown in western Canada^7^. In a previous study, we catalogued 5,000 expressed sequence tags (ESTs) accumulated during the biotrophic phase of compatible interaction between *L. culinaris* cv. Eston and an isolate of the less virulent race 1 of *C. lentis*. A total of 11 candidate effectors were mined from that EST library, and expression analysis coupled with *in planta* functional analysis revealed that most of these are *in planta* induced and likely associated with the suppression of plant immunity^8^. EST mining also identified 387 unigenes encoding proteins associated with stress and resistance responses, such as pathogenesis-related (PR) proteins and NBS-LRR R proteins. One of the resistance genes encoding a CC-NBS-LRR R protein showed differential temporal expression during the infection process with isolates of the two races in the lentil cultivar CDC Robin with partial resistance to race 1, and is likely involved in post-invasion host resistance^9^. We also identified the two effectors *CtNUDIX* and *CtToxB* through EST analysis. The expression of *CtNUDIX* triggers the biotrophy-necrotrophy switch by inducing abrupt cell death^10^ whereas *CtToxB* likely amplifies the cell death signal to accommodate the necrotrophic colonization by *C. lentis*^11^.

In this study, a transcriptomics approach was used to further dissect molecular mechanisms underlying the virulence of *C. lentis* on lentil. A cDNA plasmid library was generated and sequenced on the Sanger sequencing platform from the compatible interaction between *L. culinaris* cv. Eston and an isolate of the highly virulent race 0 of *C. lentis*. A total of 11,094 ESTs were generated and assembled into 3,488 unigenes, which were mapped onto *C. lentis* and *L. lentis* genome assemblies. As a result, 2,418 fungal and 1,070 lentil unigenes were identified. Functional annotations were assigned to fungal and plant unigenes using blastx against non-redundant protein database, UniProt database and UniProt-Gene Ontology database. This resulted in identification of 22 candidate effectors and 26 resistance genes. The expression of some of the candidate effectors and resistance genes were validated through RT-qPCR. Comparative genomics of *C. lentis* with the closely related species *C. higginsianum* revealed collinearity between genomic regions harboring candidate effectors as well as non-syntenic regions for some of the effectors, potentially due to chromosomal rearrangements, such as translocation and inversion.

## Results

### *Lens culinaris* cv. Eston x *Colletotrichum lentis* race 0 cDNA library

**Figure 1:**
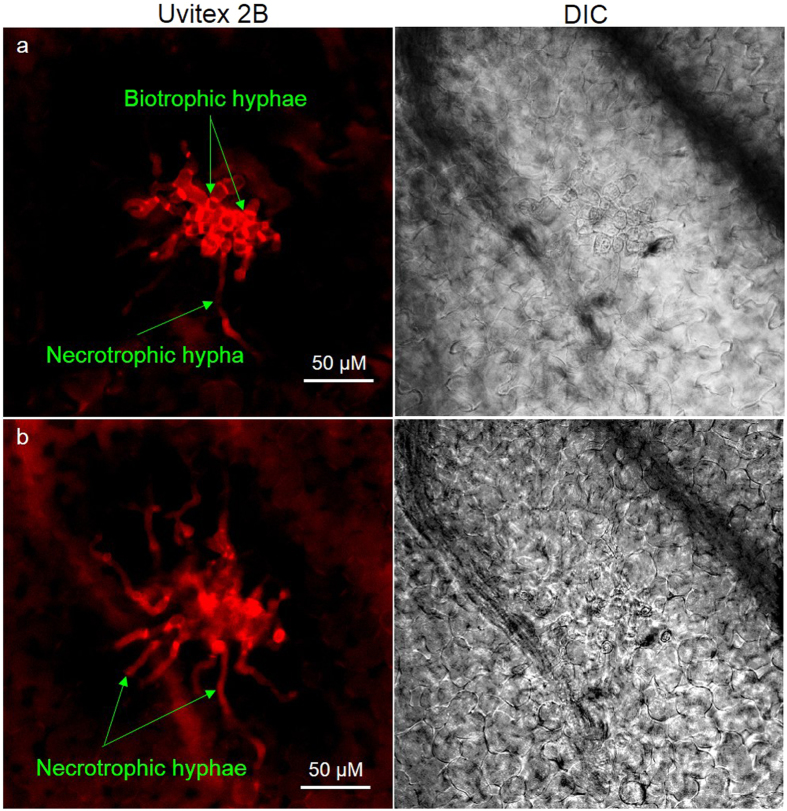
Visualization of *in planta* fungal structures of an isolate (CT-30) of the virulent race 0 of *Colletotrichum lentis* in the susceptible *Lens culinaris* cultivar Eston. Confocal laser scanning microscopy was used to scan infected plant tissues harvested at 68 hpi. (**a**) Thin secondary necrotrophic hyphae emanate from thick biotrophic hyphae; (**b**) Optical slice of image (**a**).

To capture the dynamics of transcript abundance during the onset of the symptomatic phase of *C. lentis* infection, the susceptible lentil cultivar Eston was inoculated with an isolate of the virulent race 0 of *C. lentis*. Infected tissues collected in a time-course series were visually analyzed under a confocal microscope to ensure that the pathogen was in the necrotrophic phase wherein the secondary hyphae had emanated from the biotrophic hyphae (Fig. 1a,b). This corresponded to 68 hours post-inoculation (hpi), and this infection phase was referred to as the necrotrophic phase. Poly A+ mRNA was isolated from the 68 hpi tissues and used to construct a directional cDNA plasmid library. cDNA clones were sequenced on the Sanger sequencing platform using 5′-single pass sequencing, and a total of 11,094 ESTs was generated. The average length of ESTs was 600 bp. The ESTs were assembled into 3,488 unigenes composed of 1,493 contigs and 1,895 singletons. Mapping of unigenes onto the *L. culinaris* cv. Redberry genome v0.7 assembly and the *C. lentis* genome assembly v0.9 revealed that the infection-induced transcriptome contained 30.6% plant transcripts represented by 1,070 unigenes (408 contigs and 562 singletons) and 69.4% fungal transcripts represented by 2,418 unigenes (1,085 contigs and 1,333 singletons). The average GC content of fungal unigenes was 54.9%, which was slightly higher compared to that of ESTs derived from lentil tissues infected with the less virulent race 1, whereas the GC content of plant unigenes was relatively lower at 39.64% compared to race 1 – infected tissues^8^. The fungal transcriptome of the necrotrophic phase contained nearly 20% (2,418) of the 12,086 *C. lentis* gene models estimated from the *C. lentis* genome assembly (unpublished data).

### Functional annotation of fungal and plant unigenes

**Figure 2:**
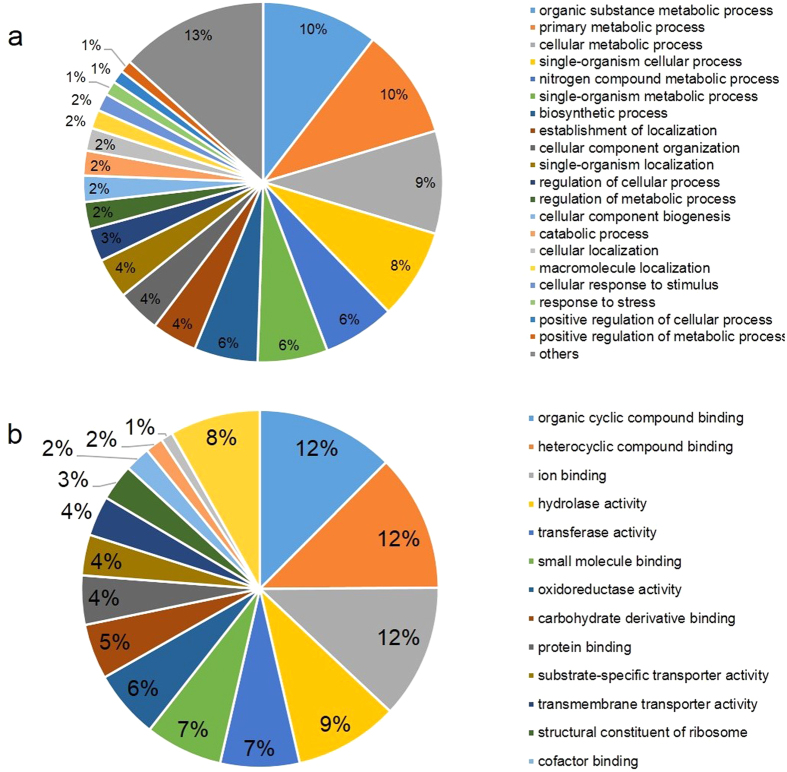
Gene ontology (GO) annotations of unigenes of an isolate (CT-30) of the virulent race 0 of *Colletotrichum lentis* at level 3 using the blast2go software. The GO terms were categorized into biological processes (**a**) and molecular functions (**b**). The pie chart slices represent the percent of unigenes identified in the particular category.

To unravel what cellular and molecular components establish necrotrophy and condition susceptibility, functional annotation was performed on plant and fungal unigenes. Fungal and plant unigenes were queried against the NCBI non-redundant protein database using blastx with an *e* value threshold of 10^−5^, and gene ontology (GO) terms were assigned to the top blast hits using the GO and UniProt databases. A total of 1,836 (75.9%) out of 2,418 fungal unigenes had blastx hits, and the GO terms were assigned to the top blastx hit of 1,500 (62.0%) unigenes. Protein domain and motifs were retrieved for 1,311 (54.3%) unigenes using InterProScan with pfam database implemented in the blast2go software^12^. Merging of GO terms retrieved through the InterProScan to existing GO annotation increased the total number of unigenes to 1,607 (66.5%) with at least one GO term, which were then organized into two ontologies based on generic terms at level 3 using the blast2go software: Biological processes and molecular functions (Fig. 2a,b, Suppl. Table 1). Biological processes were dominated by organic substance metabolic processes (10.4%), primary metabolic processes (9.8%) cellular metabolic processes (9.1%), single-organism cellular processes (8.0%), nitrogen compound metabolic processes (6.4%), single-organism metabolic processes (6.2%) and biosynthetic processes (5.7%). Molecular functions were represented by genes implicated in organic cyclic compound binding (12.5%), heterocyclic compound binding (12.4%), ion binding (12.1%), hydrolase activity (9.4%), transferase activity (7.2%), small molecule binding (7.0%), oxidoreductase activity (6.2%) and carbohydrate derivative binding (5.0%). Enzyme code annotation was performed on unigenes having GO annotations and InterProScan hits by mapping encoded proteins onto the Kyoto Encyclopedia of Genes and Genomes (KEGG) metabolic pathways. As a result, 463 enzyme code annotations were assigned to 873 unigenes and organized into 6 first level enzyme code classes: Hydrolases (208), transferases (119), oxidoreductases (83), lyases (27), isomerases (13) and ligases (13). These enzymes participated in 100 metabolic pathways, and the most populated pathways during *in planta* growth of *C. lentis* at 68 hpi was biosynthesis of antibiotics/secondary metabolites (67 enzymes) followed by purine metabolism (19 enzymes), cysteine and methionine metabolism (16 enzymes) and glycolysis/gluconeogenesis (13 enzymes). The majority of fungal unigenes (1,833 unigenes or 95%) had top blastx hits in the genus *Collectotrichum* whereas the top hit of the remaining 5% unigenes (90) were matched to *Verticillium* spp., *Trichoderma* spp., *Fusarium* spp. and others, suggesting a close phylogenetic relationship within the *Colletrichum* genus. Some of the unigenes were likely horizontally transferred from other fungal species into the genus *Colletrichum* to colonize new hosts (Suppl. Fig. 1).

**Figure 3:**
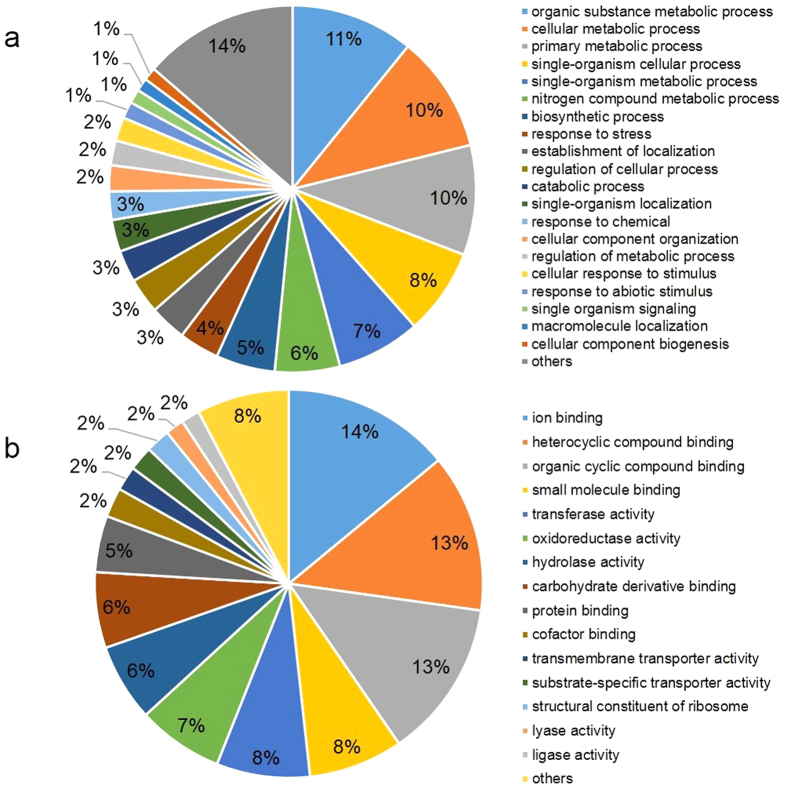
Gene ontology (GO) annotations of *Lens culinaris* cultivar Eston unigenes at level 3 using the blast2go software. The GO terms were categorized into biological processes (**a**) and molecular functions (**b**). Pie chart slices represent the percent of unigenes identified in a particular category.

Of the 1,070 plant unigenes, 800 (74.8%) had blastx hits, and the GO terms were assigned to the top blastx hit of 640 unigenes (59.8%). The InterProScan search revealed 679 unigenes (63.5%) having a pfam domain. After merging GO terms and InterProScan hits, 712 unigenes (66.5%) were assigned to one or more GO terms, which were organized into the two ontologies biological processes and molecular function based on generic terms at level 3 using the blast2go software (Fig. 3a,b, Suppl. Table 2). Biological processes comprised organic substance metabolism (10.4%), cellular metabolism (9.9%), primary metabolism (9.3), single-organism cellular processes (7.3%), single-organism metabolic processes (7.1%), nitrogen compound metabolic processes (5.5%), biosynthetic processes (5.0%) and response to stress (3.4%). Molecular functions included ion binding (14.0%) followed by heterocyclic compound binding (13.2%), organic cyclic compound binding (13.2%), small molecule binding (7.9%), transferase activity (7.8%), oxidoreductase activity (7.2%), hydrolase activity (6.5%) and carbohydrate derivative binding (6.3%). The majority of plant unigenes (731 unigenes or 90%) had top blastx hits in the Leguminosae (Suppl. Fig. 2). Unigenes having GO terms and pfam domains were searched against the KEGG database, which returned 263 KEGG Orthology identifiers assigned to 450 unigenes involved in 86 KEGG pathways. The KEGG Orthology identifiers were assigned to 6 main enzymes classes: Transferases (78), oxidoreductases (74), hydrolases (72), lyases (19), isomerases (9) and ligases (80). Comparative analysis between assigned enzyme classes to plant and pathogen unigenes revealed that the pathogen enhanced hydrolase activity (208 hydrolases) at 68 hpi, which is consistent with the onset of necrotrophy wherein the pathogen exploits these enzymes for its cell wall modification to increase the surface area and for the maceration of plant tissue to release nutrients for supporting its growth (Suppl. Fig. 3). These enzymes are involved in various pathways, such as biosynthesis of antibiotics/secondary metabolite (27 enzymes) followed by purine metabolism (15 enzymes), starch and sucrose metabolism (11 enzymes), glycolysis/gluconeogenesis (9 enzymes), citrate (TCA) cycle (8 enzymes), glutathione metabolism (7 enzymes).

### Fungal unigene mining identifies candidate effectors

**Figure 4:**
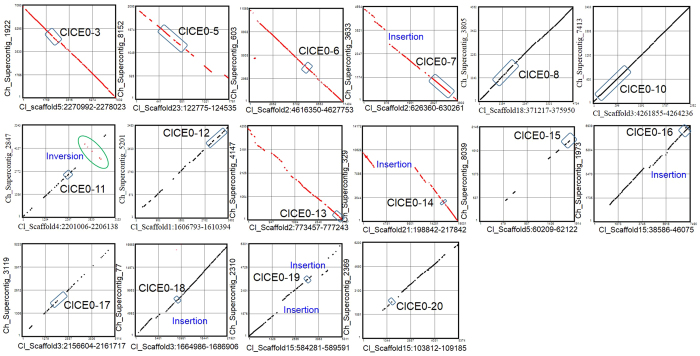
Comparative genomics. Genomic regions harboring candidate effectors were extracted from the *Colletotrichum lentis* and the closely related *C. higginsianum* genomes. Synteny dot plots were generated with a sliding window of 30 nucleotides with 5 mismatches and 50% identity. Black and red diagonal lines represent pairwise forward-forward and forward-reverse alignments, respectively. Mapped boxes indicate candidate effector coordinates.

Cellular components derived from the GO term analysis of *C. lentis* unigenes at level 2 revealed 36 unigenes of extracellular region and extracellular region part (Suppl. Fig. 4). Those having less than 300 aa and a N-terminal signal peptide, and lacking a transmembrane helix and a glycosylphosphatidylinisotol (GPI) anchor addition site were considered as candidate effectors. This reduced the number to 22, including 1 lineage-specific effector (ClCE0-21; Table 1), representing 6% of the candidate effectors identified in the *C. lentis* genome. Thirteen of these were mapped onto 4 scaffolds of the *C. lentis* genome assembly (unpublished data). Three candidate effectors (ClCE0-1, ClCE0-15 and ClCE0-16) are located within 10 Kb of scaffold ends of the *C. lentis* genome, suggesting their sub-telomeric origin, which was one of the criteria used by Syme and colleagues^13^ to identify effectors. Comparative genomics was used to examine the collinearity of genomic regions of *C. lentis* harboring candidate effectors with the closely related species *C. higginsianum* for potential genomic rearrangements. Homologous genomic regions flanking the candidate effectors were retrieved from the *C. lentis* (unpublished) and *C. higginsianum*^14^ genome assemblies and pair-wise aligned. As a result, microsyntenic blocks were identified for candidate effectors except ClCE0-1 to 2, ClCE0-4, ClCE0-9, ClCE0-21 and ClCE0-22 based on 30% sequence identity in a sliding window of 30 nucleotides with 5 mismatches. Insertions ranging from ~50 bp (ClCE0-7) to ~7,125 bp (ClCE0-14) were detected in the syntenic blocks. Multiple insertion events were identified in the flanking sequences of the candidate effectors ClCE0-7, ClCE0-13, ClCE0-16, ClCE0-18 and ClCE0-19. In addition, a ~1,200 bp translocation was mapped onto the syntenic block of candidate effector ClCE0-11 (Fig. 4).

Disulfide bonding between cysteine residues confers stability to effectors upon their secretion into the apoplast, and effectors either lacking or possessing few cysteine residues are likely translocated into host plant cells wherein they interfere with pathogen-associated molecular pattern-triggered immunity or effector-triggered immunity to condition susceptibility. The number of cysteine residues in candidate effectors varied from 1 (ClCE0-17) to 24 (ClCE0-16). Six candidate effectors lacked the cysteine residue (ClCE0-1, ClCE0-10, ClCE0-12 to 14, ClCE0-18), suggesting that these proteins along with those having few residues might translocate into host cells (Table 1). We further scanned candidate effectors for potential N- and O-glycosylation sites, which render the binding of sugar to asparagine, and serine and threonine residues, respectively. Heavily glycosylated proteins are unlikely to be secreted into hosts but attach to the fungal cell membrane or cell wall following the carbohydrate binding. No glycosylation site was detected in six of the candidate effectors (ClCE0-6, ClCE0-8, ClCE0-10, ClCE0-15, ClCE0-20 and ClCE0-21) and two (ClCE0-1 and ClCE0-5) contained a single glycosylation site each. Heavy glycosylation was observed in 8 candidate effectors: ClCE0-3 (56 sites), ClCE0-18 (52 sites), ClCE0-11 (30 sites), ClCE0-12 (19 sites), ClCE0-16 (16 sites), ClCE0-7 (13 sites), ClCE0-4 (11 sites) and ClCE0-13 (10 sites). These proteins are unlikely to be translocated into host cells. The lineage specific candidate effector ClCE0-21 contained 6 cysteine residues, which are likely to form 3 disulfide bridges, and lacked both N- and O- glycosylation sites, suggesting that the effector probably exerts its activity in the apoplast.

The 21 candidate effectors had blastx hits in the genus *Colletotrichum*. Ten of them lacked functional annotation in the NCBI non-redundant database whereas the remaining 11 were annotated as prp 4 c domain-containing protein (ClCE0-3), intracellular hyphae protein 1 (ClCE0-4), ToxB (ClCE0-5), cutinase (ClCE0-6), GDSL-like lipase/acylhydrolase (ClCE0-7 and 14), cerato-platanin (CP, ClCE0-8), cyclin-dependent protein kinase regulator pho80 (ClCE0-10), sperm coat protein (SCP)-like extracellular protein (ClCE0-13), cyanovirin-N domain containing protein (ClCE0-15) and CHD5 domain-containing protein (ClCE0-17).

**Figure 5:**
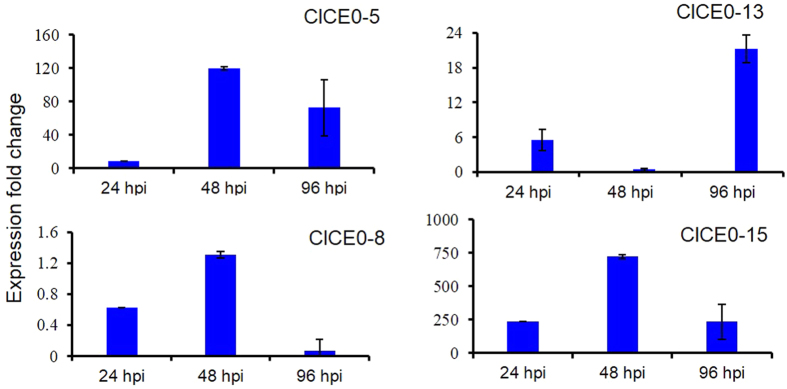
Expression profiling of candidate effectors of an isolate (CT-30) of the virulent race 0 of *Colletotrichum lentis* in *Lens culinaris* cultivar Eston. Relative expression was determined using the comparative C_T_ method (Livak and Schmittgen^48^) with ΔCT value obtained for vegetative hyphae as a calibrator in 3 *in planta* time-points: 24 hpi (appressorium penetration), 48 hpi (biotrophic phase) and 96 hpi (necrotrophic phase). The actin gene was used as a reference gene.

Both, *ToxB* and *CP* transcripts were also present in the *C. lentis* race 1-infected lentil transcriptome and through RNAi and heterologous expression, we demonstrated their role as virulence factors^8^,^11^. *ToxB* expression in Eston inoculated with an isolate of race 0 peaked at 48 hpi, with a 120-fold increase in transcript accumulation compared to *in vitro* mycelia, which was reduced to 73-fold expression at 96 hpi. In contrast, *CP* expression remained at a basal level (constitutive expression) (Fig. 5). ClCE0-4 contains two lysine motifs (LysM), 7 cysteine residues and 11 glycosylation sites, and is homologous to the *C. lindemuthianum* glycoprotein CIH1 (39% amino acid identity)^15^. The two LysM are likely to sequester *C. lentis* chitin oligosaccharides released by plant chitinases (PR4) and hence camouflage the pathogen as was reported in other pathogens^16^. Similar to ClCE0-4, the ClCE0-15 is a cysteine-rich protein (11 residues) that binds to *N*-acetylglucosamine-containing carbohydrates, such as chitin, chitio-oligosaccharides and peptidoglycan^17^. It contains a CyanoVirin-N homology domain (pfam08881) and lacks a glycosylation site. *ClCE0-15* expression peaked at 48 hpi in Eston inoculated with an isolate of race 0, with a 722-fold increase in transcript accumulation compared to *in vitro* mycelia though this was reduced to a 238-fold increase at 96 hpi (Fig. 5). The presence of cutinase in the transcriptome suggests its involvement in pathogenesis beyond the penetration stage and likely in carbon acquisition as reported for *Venturia inaequalis*^18^. Interestingly, two paralogous unigenes, ClCE0-7 and ClCE0-14 encoded GDSL-like lipase/acylhydrolase (GDSL) whereas the unigene ClCE0-13 encoded a SCP-like extracellular protein and is homologous to the plant pathogenesis-related protein 1 (PR1). Similar to *PR1*, the *ClCE0-13* was induced *in planta* following infection of Eston with a race 0 isolate (Fig. 5), as was also reported for *Moniliophthora perniciosa*, the causal agent of witches’ broom disease in Cacao^19^.

### Plant unigene mining identifies regulators of plant immunity

Biological processes derived from the GO term analysis of lentil unigenes at level 2 revealed 11 and 138 unigenes implicated in immune system processes and response to stimulus, respectively (Suppl. Fig. 5). All 149 sequences were manually analyzed to retrieve genes involved in eliciting plant defense responses, and those involved in recognition of fungal effectors, signalling of pathogen perception, altering phytohormone level, transcribing defense response genes were considered as regulators of defense. As a result, the number of resistance genes was reduced to 26 unigenes (hereafter referred to as resistance genes). Of these, 25 had top blastx hits in legume species, such as *Medicago truncatula, Cicer arietinum, Glycine max* and *G. soja*, suggesting a conserved resistance mechanism across legume species (Table 2). Among them are suppressor of NPR1-1, constitutive 1 (*Snc1*), *PR1, PR4, PR5, PR10 and LcDirigent*.

**Figure 6:**
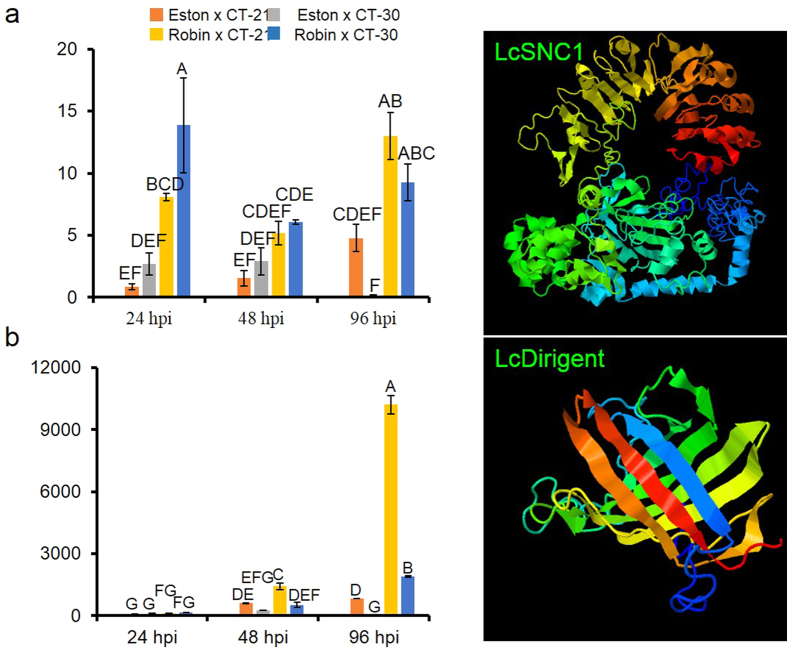
Expression profiling of resistance genes *LcSNC1* encoding a NBS-LRR class resistance protein suppressor of npr1-constitutive 1 (**a**), and *LcDirigent* (**b**) in *Lens culinaris* cultivars Eston (susceptible to both races) and CDC Robin (partially resistant to race 1) following infection by isolates of race 0 (CT-30) and race 1 (CT-21) of *Colletotrichum lentis*. Relative expression was determined using the comparative C_T_ method (Livak and Schmittgen^48^) with ΔCT value obtained for mock-inoculated genotype as a calibrator in 3 *in planta* time-points: 24 hpi, 48 hpi and 96 hpi. The actin gene was used as a reference gene. Bars with same letters are not significantly different in expression. Three-dimensional structural models of resistance proteins are shown next to their respective expression charts.

The *Snc1* (LcCT-30-21) was induced significantly in the partially resistant lentil cultivar CDC Robin compared to the susceptible cultivar Eston challenged with isolates of races 0 and 1 at 24 hpi (biotrophic phase) and 96 hpi (necrotrophic phase; *p* < 0.05) (Fig. 6a), suggesting that the salicylic acid (SA)-dependent defense responses as evident by the presence of *PR1* and *PR5* in the transcriptome are activated during fully and partially compatible interaction. Blastp search of LcSnc1 resulted in the identification of homologs conserved among the members of the Leguminosae, such as *Pisum sativum, Phaseolus vulgaris, Cicer arietinum, Glycine max, G. soja, Medicago truncatula, Cajanus cajan, Arachis duranensis, A. ipaensis, Vigna radiata* and *V. angularis*. The overall mean diversity in the entire population was 0.478 whereas the mean diversity within a subpopulation representing the LcSnc1 clade with 13 members belonging to *M. truncatula, C. arietinum* and *L. culinaris* was 0.259 (25.9% sequence divergence), suggesting a high level of conservation among the Snc1 homologs (Suppl. Fig. 6). Five unigenes (LcCT-30-1, LcCT-30-2, LcCT-30-11, LcCT-30-14 and LcCT-30-15) encoded chitinase hevein PR4 proteins that are indicative of the jasmonic acid (JA)- dependent defense response. Two PR10 proteins (Bet v1 [LcCT-30-4] and DRR49 [LcCT-30-5]) were also found in the transcriptome. The major pollen allergen (Bet v1) protein was identified from white birch pollen and induced in response to pathogen challenge^20^. The DRR49 protein was identified and induced in pea following the challenge with the non-host pathogen *Fusarium solani* f. sp. *phaseoli*^21^. In addition, we identified a highly expressed unigene, *LcCT-30-3*, which was induced 1,416-fold at 48 hpi and 10,224-fold at 96 hpi in the lentil cultivar CDC Robin with partial resistance to race 1 after challenge with a race 1 isolate whereas inoculation with a race 0 isolate reduced the transcript accumulation to 520-fold at 48 hpi and 1,890-fold at 96hpi (Fig. 6b). The protein encoded by this unigene contains a dirigent domain (pfam03018), hence called LcDirigent, and shows homology to pea disease resistance response protein DRR206 (85% identity). It is a small protein of 184 amino acid residues with a molecular weight of 19.23 KDa that follows the secretory pathway as it contains a N-terminal signal peptide (Fig. 6b). No sequence diversity was observed between the susceptible cultivars Eston and the partially resistant cultivar CDC Robin, whereas 2 synonymous and 2 non-synonymous single nucleotide polymorphism variants were identified in the *LcDirigent* homolog of *L. ervoides* (1.1% sequence divergence at the protein level; Suppl. Fig. 7). The LcDirigent homologs are conserved across the plant kingdom, and the top 74 blastp hits with an *e* value of 10^−6^ showed 24.3% divergence whereas legume homologs exhibited 21.4%, suggesting a balancing selection operating on dirigent genes to maintain conserved disease resistance response against microbial pathogens.

## Discussion

The outcome of plant-pathogen interactions relies on a complex interplay between plant disease resistance proteins and fungal effectors, and goes in favour of adapted pathogens as they either successfully evade the plant’s innate immunity and/or exploit it to facilitate their infection processes. To investigate the complex interplay, we applied a EST-based transcriptomics approach to the compatible *Lens culinaris* - *C. lentis* pathosystem. The presence of 2,418 *C. lentis* gene models in the transcriptome suggests that the pathogen allocates a substantial proportion of the genetic program, an estimated 20% of the gene models, to cause anthracnose on lentil. Among them are 22 candidate effectors that are reported to be compartmentalized in the dynamic regions of the genome, such as gene-sparse blocks, subtelomeric regions, pathogenicity islands or conditionally dispensable chromosomes (Table 1)^22^. Plasticity of such compartments enables genomic rearrangements, such as translocation, inversion, duplication and insertion/deletion, leading to the emergence of lineage-specific regions enriched in virulence effectors as was reported in the vascular wilt pathogen *Verticillium dahliae*^23^. Three of the candidate effectors (ClCE0-1, ClCE0-15 and ClCE0-16) are likely located at the chromosome ends. Such subtelomeric effectors tend to evolve at a higher rate than the rest of the genome as was reported for the *Magnaporthe oryzae* effector gene *Avr-Pita*, which is localized 48 bp from the telomere on chromosome 3^24^,^25^,^26^, and their diversification triggers a collapse of the plant detection system, especially when mediated by the NBS-LRR R receptors. Furthermore, comparative genomics revealed local genomic rearrangements, such as insertion/deletion and inversion between genomic regions harbouring candidate effectors of *C. lentis* and their orthologs in *C. higginsianum*, suggesting that such spatial variations in the genomes led to different niche colonization (Fig. 4).

In addition to two previously characterized virulence effectors ToxB and CP^8^,^11^, we identified 9 effectors with functional homologs in the genus *Colletotrichum*. These include prp 4 c domain-containing protein, CIH1, cutinase, GDSL-like lipase/acylhydrolase, cyclin-dependent protein kinase regulator pho80, SCP-like extracellular protein, cyanovirin-N and CHD5 domain-containing protein (Table 1). Both CIH1 and cyanovirin-N sequester chitin oligosaccharides released by plant chitinases and hence facilitate the evasion of pathogen detection^16^,^17^. This suggests that the host is responding to pathogen attack in cells immediately surrounding the proliferating pathogen. Cutinases are known to hydrolyze cutin monomers, building blocks of the first layer of physical defense (cuticle) and homologs are conserved across the kingdom fungi^27^,^28^. The role of cutinase as a virulence factor was recently confirmed by RNAi in the phytopathogen *C. truncatum*, the causal agent of anthracnose on chilli^29^. However, the presence of cutinase in the transcriptome at 96 hpi suggests its potential involvement in carbon acquisition as reported for *Venturia inaequalis*^18^. Interestingly, three unigenes (ClCE0-7 and ClCE0-14, and ClCE0-13) encoding proteins similar to plant disease resistance proteins, GDSL-like lipase/acylhydrolase and SCP-like extracellular protein were also identified in the transcriptome. GDSL-like lipase/acylhydrolase is well characterized in *Arabidopsis thaliana* and regulates ethylene-dependent local and systemic resistance against *Alternaria brassicicola, Erwinia carotovora* and *Pseudomonas syringae*^30^ though the role of fungal GDSL remains unknown. SCP-like extracellular protein contains a single SCP/Tpx-1/Ag5/PR-1/Sc7 (SCP/TAPS, smart00198), and is homologous to the plant PR1 protein. The SCP/TAPS may have an antimicrobial activity similar to the plant PR1, which could provide the pathogen with a competitive advantage by limiting the growth of others^31^,^32^,^33^.

Plant unigene mining identified 26 disease resistance genes implicated in recognition of fungal effectors, signalling of pathogen perception, altering phytohormone level and transcribing defense response genes. Nevertheless, *C. lentis* exploits some of the lentil defense responses to support its necrotrophic lifestyle (Table 2). The Snc1 (Fig. 6a), a TIR-NBS-LRR class of highly conserved resistance protein (25.9% sequence divergence among legume species) interacts with the non-expressor of PR1 protein, a receptor of SA, and its presence in the transcriptome suggests that the SA-dependent defense responses as evident by the presence of marker genes *PR1* and *PR5* in the transcriptome are activated. However, although the pathogen exploits *Snc1*-mediated defense responses during the symptomatic necrotrophic infection where it requires dead or necrotising plant tissues, it remains unclear how the pathogen copes with such host plant responses during the asymptomatic biotrophic infection phase (Fig. 6a). Similar to *Arabidopsis thaliana, Snc1* in lentil is mapped onto a cluster of a paralogous TNL class of resistance genes in the *L. culinaris* genome. A gain-of-function allele of *Snc1* triggers constitutive expression of SAR markers, such as PR1 (LcCT-30-23), PR2 and PR5 (LcCT-30-24 to LcCT-30-26) and enhances resistance to *Pseudomonas syringae* pv. *maculicola* and *Peronospora parasitica*^34^. As no growth penalty was observed in the *Snc1* mutant, mining allelic diversity of *Snc1* in wild relatives of lentil, such as *L. ervoides* may be useful as targets for introgressing constitutive expression of plant defense in cultivars without compromising the yield potential as was reported in overexpression of other *R* genes. Interestingly, unigenes encoding the chitinase hevein PR4 proteins are also found in the transcriptome (Table 2). The PR4 proteins are secreted into the plant apoplast upon pathogen perception wherein they directly hydrolyze chitin, a main constituent of the fungal cell wall. Similarly, PR1 and PR5 are also induced during pathogen attack and mobilized to the apoplast wherein they exert their antifungal activity. However, in contrast to PR1 and PR5, mobilization of chitinases into the apoplast to counter pathogen attack is a JA- dependent plant defense response, which is effective against necrotrophic pathogens, suggesting a complex interplay between SA- and JA-responsive defense pathways during the compatible lentil-*C. lentis* interaction. Similar to PR proteins, the highly expressed LcDirigent (LcCT-30-3) is also transported to the apoplast wherein it polymerizes monolignols to form lignans. As a result, the plant cell wall is lignified and thereby strengthened to prevent further spread of the pathogen. To counter cell wall reinforcement, the pathogen delignifies lignocellulose using lignin peroxidases that trigger compatibility. Assuming interspecific functional diversity in LeDirigent from *L. ervoides* against race 0 isolates, *LeDirigent* alleles could present a target for introgression into the cultivated genepool.

Unlike other PR proteins that are either extracellular or localized in the vacuole, PR10 proteins (Bet v1 [LcCT-30-4] and DRR49 [LcCT-30-5]) lack signal peptides and are localized in the intracellular space. Bet v1 and DRR49 are putative RNases^35^,^36^,^37^, but LcCT-30-4 and LcCT-30-5 lack nuclear localization signals and are therefore unlikely to enter into the host nucleus as was reported in case of DRR49 from pea^21^. The role of DRR49 was demonstrated in transgenic potato in the form of enhanced resistance to early dying disease caused by *Verticillium dahliae*^38^. It is likely that both proteins are synthesized in response to pathogen attack and released from the damaged tissues to directly act on the pathogens^39^.

To summarize, we sequenced the *C. lentis*-infected lentil transcriptome consisting of 11,094 ESTs representing 2,418 unigenes of fungal, and 1,070 unigenes of plant origin. Functional annotation of unigenes revealed a complex molecular interplay between *L. culinaris* and *C. lentis* during the compatible interaction as is evident from the presence of 26 resistance genes and 22 effector genes in the transcriptome. The resistance genes include both negative and positive regulators of plant immunity, such as *Snc1* and *dirigent*, as well as the markers of antagonistic defense signalling pathways, such as PR1 and PR5 (SA) and PR4 (JA). The regulatory cross-talk between the signalling pathways allows plants to trigger defense responses specialized in tackling various groups of pathogens, such as biotrophic and necrotrophic pathogens. *Colletotrichum lentis* employs a hemibiotrophic infection strategy to colonize lentil, hence the susceptibility of the lentil cultivar Eston could be attributed to the fact that *C. lentis* exploits phytohormone cross-talk by employing effectors (ETS, Effector triggered susceptibility). Genomic analysis revealed three sub-telomeric effectors whose diversification may lead to ETS. Intraspecific molecular diversity and functional characterization of the effectors will clarify their role in the susceptibility of lentil.

## Methods

### Plant and fungal material

Lentil (*L. culinaris*) plants of Canadian cultivars Eston with complete susceptibility to both races of *C. lentis*, and CDC Robin with partial resistance to race 1 were grown in 4 inch pots containing commercial potting mixture as described previously^9^. Isolates CT-30 (race 0) and CT-21 (race 1) of *C. lentis* were routinely maintained on oat-meal agar (OMA) plates containing 30 g blended quick oats (The Quaker Oats Company, Chicago, USA), 8.8 g agar (Becton, Dickinson and Company, Sparks, USA), and 1 L deionized water supplemented with 0.01% chloramphenicol (AMRESCO, Solon, USA). For infection assays, conidia were collected from 10-day-old OMA plates by flooding the sterile water.

### cDNA library construction and Sequencing

Leaflets were detached from 21 days old Eston plants and inoculated in Petri dishes lined with wet filter paper with droplets of conidial suspension (5 × 10^4^ conidia/mL) of CT-30. The progress of infection was microscopically assessed, and plant tissues were harvested by punching the infection sites when the fungus switched to the necrotrophic phase. Samples were flash-frozen in liquid nitrogen. Total RNA was isolated from the harvested tissues using the LiCl extraction method^40^, and mRNA was purified from the total RNA using PolyATtract^®^ mRNA isolation system IV (Promega, Madison, USA) following the manufacturer’s protocol.

Five μg poly(A)+ RNA (mRNA) was used to generate a cDNA plasmid library using the pBluescript^®^ II XR cDNA library construction kit (Stratagene, La Jolla, USA) following the manufacturer’s instructions. The resultant recombinant plasmids were used to transform XL10-Gold ultracompetent cells (Stratagene, La Jolla, USA). cDNA clones were sequenced on the Sanger sequencing platform using M13 reverse universal primer at the DNA Technology Unit, National Research Council of Canada, Saskatoon, Canada.

### Bioinformatics

ESTs were scanned for pBluescript vector and adaptor (GAATTCGGCACGGGAGG) contamination using VecScreen (http://ncbi.nlm.nih.gov/VecScreen), which were clipped using a custom AWK script. SeqClean script (http://sourceforge.net/projects/seqclean) was used to remove any residual vector or adapter sequence, and ESTs with less than 75 bp were removed from further analysis. ESTs were mapped onto the *C. lentis* v0.9 and *L. culinaris* v0.7 genome assemblies using blastn implemented in BioEdit v7.2.5 41. Plant and fungal ESTs were assembled into unique sequences (unigenes) using Sequencher v4 (Gene Codes, Ann Arbor, USA). Open reading frames were retrieved from unigenes using ORF-Predictor^42^. Unigenes were functionally annotated using the blastx command implemented in BLAST stand-alone with the non-redundant protein database^43^. Gene ontology terms were assigned to unigenes using the blast2go software. In addition, unigenes were also searched against the KEGG and InterPro databases^44^,^45^.

SignalP v4.1 (http://cbs.dtu.dk/services/SignalP) and WoLF PSORT^46^ were used to predict the N-terminal signal peptide in proteins (≤300 aa) encoded by fungal unigenes. Transmembrane helices and GPI anchor addition sites were predicted using TMHMM v2 (http://cbs.dtu.dk/services/TMHMM) and PredGPI (http://gpcr.biocomp.unibo.it/predgpi). NetNGlyc v1 (http://cbs.dtu.dk/services/NetNGlyc) and NetOGlyc v2 (http://cbs.dtu.dk/services/NetOGlyc) were used to predict N- and O- glycosylation sites. Genomic regions harboring the candidate effectors were retrieved using SAMtools^47^, and DNAMAN v5.2.2 (Lynnon Corporation, Pointe-Claire, Canada) was used to generate synteny plots.

### Infection time-course of cDNA library and RT-qPCR

Three-week-old Eston and CDC Robin plants were spray-inoculated with *C. lentis* isolates CT-30 (race 0) and CT-21 (race 1) conidia (5 × 10^4^ conidia/mL). The progress of infection was microscopically evaluated. Three *in planta* time-points were selected for expression profiling: 24 hpi (appressorium penetration), 48 hpi (biotrophic phase) and 96 hpi (necrotrophic phase). Infected tissues were harvested from three biological repeats and flash-frozen in liquid nitrogen. Total RNA was extracted using the SV Total RNA Isolation System (Promega Corporation, Fitchburg, USA) following the manufacturer’s instruction. In addition, in-column DNase treatment was also performed during the RNA extraction. First strand cDNA was synthesized from 1 μg DNase-treated total RNA using the SensiFAST™ cDNA Synthesis Kit (Bioline, London, UK) and diluted to 10-fold in UltraPure DNase/RNase free-distilled water (Life Technologies, Cergy Pontoise, France).

Expression profiling was performed on the Bio-Rad Real-Time PCR detection platform CFX-384 (Bio-Rad, Hercules, USA). *Lens culinaris* and *C. lentis* actin genes were used as reference genes to normalize the expression of target genes. The 10 μL reaction contained 5 μL of 2x SensiFAST™ SYBR^®^ (Bioline, London, UK), 400 nM each of forward and reverse primers and 2 μL cDNA as template. A three-step thermal cycling (2 min pre-heating at 95 °C followed by 39-cycles of 5 sec at 95 °C, 10 sec at 60 °C and 20 °C sec at 72 °C) was performed to amplify reference and target genes. A melt curve step was also incorporated to detect any potential non-specific amplicons and primer dimer. The relative expression of target genes was calculated using the comparative CT method^48^ using a ΔCT value obtained for vegetative hyphae or mock inoculated genotypes as a calibrator. All relative expression values of genes were reported as means ± standard errors of the means on a 2-log scale. Primers used in RT-qPCR analyses are listed in Supplementary file 3.

### Phylogeny and 3D modeling of resistance genes

Homologous sequences were retrieved from the non-redundant protein database using an *e* value of 1e-30 and parsed using a custom AWK script. Non-rooted phylogenetic trees were constructed using RAxML^49^. Three dimension structural models of resistance proteins were generated through the I-TASSER portal^50^.

## Additional Information

**How to cite this article**: Bhadauria, V. *et al*. Transcriptome analysis reveals a complex interplay between resistance and effector genes during the compatible lentil-*Colletotrichum lentis* interaction. *Sci. Rep.* **7**, 42338; doi: 10.1038/srep42338 (2017).

**Publisher's note:** Springer Nature remains neutral with regard to jurisdictional claims in published maps and institutional affiliations.

## Supplementary Material

Supplementary Information

## Figures and Tables

**Table 1:** List of candidate effectors identified during the compatible interaction of an isolate of the virulent race 0 of *Colletotrichum lentis* with the susceptible *Lens culinaris* cultivar Eston.

Effectors	Accessions	Size (aa)	Cysteines	N-Gly	O-Gly	Functional annotation	Organism	e value
ClCE0-1	JZ923043	75	0	0	1	GLRG_10010	*Colletotrichum graminicola*	1e-15
ClCE0-2	JZ923044	188	6	2	1	CH063_00402	*Colletotrichum higginsianum*	2e-78
ClCE0-3	JZ923045	247	14	1	55	prp 4 c domain-containing protein	*Colletotrichum orbiculare*	5e-73
ClCE0-4	KY502250	168	7	0	11	Intracellular hyphae protein 1	*Colletotrichum higginsianum*	5e-74
ClCE0-5	JZ923047	94	5	0	1	ToxB protein	*Colletotrichum higginsianum*	2e-22
ClCE0-6	KY502251	239	5	0	0	Cutinase	*Colletotrichum higginsianum*	7e-77
ClCE0-7	JZ923049	263	3	1	12	GDSL-like Lipase/Acylhydrolase	*Colletotrichum higginsianum*	2e-138
ClCE0-8	JZ923050	138	4	0	0	Cerato-platanin	*Colletotrichum higginsianum*	5e-74
ClCE0-9	JZ923051	132	4	1	3	CSUB01_09068	*Colletotrichum sublineola*	5e-60
ClCE0-10	JZ923052	197	0	0	0	Cyclin-dependent protein kinase regulator pho80	*Colletotrichum orbiculare*	4e-73
ClCE0-11	JZ923053	195	10	3	27	CH063_08487	*Colletotrichum higginsianum*	3e-36
ClCE0-12	JZ923054	261	0	0	19	CH063_11932	*Colletotrichum higginsianum*	2e-30
ClCE0-13	JZ923055	223	0	0	10	SCP-like extracellular protein	*Colletotrichum fioriniae*	2e-36
ClCE0-14	JZ923056	260	0	2	5	GDSL-like Lipase/Acylhydrolase	*Colletotrichum higginsianum*	5e-120
ClCE0-15	JZ923057	121	11	0	0	Cyanovirin-N domain containing protein	*Colletotrichum higginsianum*	4e-35
ClCE0-16	JZ923058	234	24	0	16	CH063_06801	*Colletotrichum higginsianum*	7e-61
ClCE0-17	JZ923059	231	1	1	3	CHD5 domain-containing protein	*Colletotrichum higginsianum*	3e-137
ClCE0-18	JZ923060	189	0	2	50	CSUB01_07212	*Colletotrichum sublineola*	2e-04
ClCE0-19	JZ923061	76	8	0	5	CGGC5_10188	*Colletotrichum gloeosporioides*	2e-18
ClCE0-20	JZ923062	85	8	0	0	CGGC5_1257	*Colletotrichum gloeosporioides*	1e-18
ClCE0-21	JZ923063	122	6	0	0	Lineage specific	*Colletotrichum lentis*	0
ClCE0-22	JZ923064	86	8	1	1	CH063_01644	*Colletotrichum higginsianum*	2e-04

**Table 2:** List of resistance genes of *Lens culinaris* cultivar Eston expressed following infection with an isolate of the virulent race 0 of *Colletotrichum lentis*.

Unigenes	Accessions	Functional annotation	Organism	e value	InterPro motif
LcCT-30-1	JZ923065	Chitinase Hevein PR-4 Wheatwin2	*Medicago truncatula*	2e-88	Barwin domain
LcCT-30-2	JZ923066	Chitinase Hevein PR-4 Wheatwin2	*Medicago truncatula*	7e-100	Barwin domain
LcCT-30-3	JZ923067	Dirigent	*Glycine max*	5e-111	Resistance response protein
LcCT-30-4	JZ923068	Pathogenesis-related bet v 1 family	*Medicago truncatula*	7e-102	Bet v I type allergen
LcCT-30-5	JZ923069	Disease-resistance response DRR49	*Medicago truncatula*	3e-104	—
LcCT-30-6	JZ923070	ABA-responsive protein ABR17	*Medicago truncatula*	10e-84	Bet v I type allergen
LcCT-30-7	JZ923071	Dihydroflavonol 4-reductase	*Medicago truncatula*	3e-108	NAD-dependent epimerase/dehydratase
LcCT-30-8	JZ923072	Regulator of Vps4 activity in the MVB pathway	*Medicago truncatula*	2e-143	Vacuolar protein sorting Ist1
LcCT-30-9	JZ923073	Jasmonate zim-domain	*Medicago truncatula*	3e-116	—
LcCT-30-10	JZ923074	Glycolipid transfer protein	*Medicago truncatula*	3e-122	Glycolipid transfer protein domain
LcCT-30-11	JZ923075	Chitinase (Class I) Hevein	*Medicago truncatula*	4e-31	—
LcCT-30-12	JZ923076	Calcium-binding protein CML38	*Cicer arietinum*	3e-29	EF-hand domain
LcCT-30-13	JZ923077	LURP-one-related 10-like protein	*Medicago truncatula*	8e-102	LURP1-related protein domain
LcCT-30-14	JZ923078	Class I chitinase	*Medicago sativa*	1e-82	Chitin-binding, type 1
LcCT-30-15	JZ923079	Chitinase Hevein PR-4 Wheatwin2	*Medicago truncatula*	2e-78	Barwin domain
LcCT-30-16	JZ923080	LURP-one-related 10-like protein	*Medicago truncatula*	8e-66	LURP1-related protein domain
LcCT-30-17	JZ923081	SBP (S-ribonuclease-binding) family protein	*Medicago truncatula*	4e-59	—
LcCT-30-18	JZ923082	Serine threonine- kinase HT1-like	*Gossypium raimondii*	3e-42	—
LcCT-30-19	JZ923083	Dihydroflavonol 4-reductase flavanone 4-reductase	*Glycine soja*	3e-42	NAD(P)-binding domain
LcCT-30-20	JZ923084	ABA-responsive protein	*Medicago truncatula*	1e-17	Bet v 1 type allergen
LcCT-30-21	JZ923085	Suppressor of npr1-1, Constitutive 1 (NBS-LRR)	*Medicago truncatula*	1e-40	Leucine-rich repeat domain
LcCT-30-22	JZ923086	WRKY family transcription factor	*Medicago truncatula*	3e-10	Barwin domain
LcCT-30-23	JZ923087	CAP, cysteine-rich secretory, antigen 5	*Medicago truncatula*	3e-85	—
LcCT-30-24	JZ923088	Pathogenesis-related thaumatin family	*Medicago truncatula*	6e-91	Thaumatin
LcCT-30-25	JZ923089	Pathogenesis-related thaumatin family	*Medicago truncatula*	8e-142	Thaumatin
LcCT-30-26	JZ923090	Pathogenesis-related thaumatin family	*Medicago truncatula*	9e-79	Thaumatin

## References

[b1] FAOSTAT Available at: http://faostat3.fao.org (*Accessed:* 25^th^ May, 2016).

[b2] Morrall R. A. A. & Pedersen E. A. Discovery of lentil anthracnose in Saskatchewan in 1990. Can. Plant Dis. Surv. 71, 105–106 (1991).

[b3] Chongo G., Bernier C. C. & Buchwaldt L. Control of anthracnose in lentil using partial resistance and fungicide applications. Can. J. Plant Pathol. 21, 16–22 (1999).

[b4] Jones J. D. G. & Dangl J. L. The plant immune system. Nature 444, 323–329 (2006). 17108957 10.1038/nature05286

[b5] Dawkins R. The Extended Phenotype: The Long Reach of the Gene. (Oxford University Press, 1999).

[b6] Raffaele S. & Kamoun K. (2012) Genome evolution in filamentous plant pathogens: Why bigger can be better. Nature Rev. Microbiol. 10, 417–430 (2012). 22565130 10.1038/nrmicro2790

[b7] Buchwaldt L., Anderson K. L., Morrall R. A. A., Gossen B. D. & Bernier C. C. Identification of lentil germplasm resistant to *Colletotrichum truncatum* and characterization of two pathogen races. Phytopathology 94, 236–43 (2004). 18943971 10.1094/PHYTO.2004.94.3.236

[b8] Bhadauria V., Banniza S., Vandenberg A., Selvaraj G. & Wei Y. EST mining identifies proteins putatively secreted by the anthracnose pathogen Colletotrichum truncatum. BMC Genom. 12, 327 (2011). 10.1186/1471-2164-12-327 PMC3149586 21699715

[b9] Bhadauria V., Banniza S., Vandenberg A., Selvaraj G. & Wei Y. Overexpression of a novel biotrophy-specific *Colletotrichum truncatum* effector CtNUDIX in hemibiotrophic fungal phytopathogens causes incompatibility with their host plants. Eukaryot Cell 12 **(1)**, 2–11 (2013). 22962277 10.1128/EC.00192-12 PMC3535838

[b10] Bhadauria V. . Identification of *Lens culinaris* defense genes responsive to the anthracnose pathogen *Colletotrichum truncatum*. BMC Genet. 14, 31 (2013). 23631759 10.1186/1471-2156-14-31 PMC3666911

[b11] Bhadauria V., MacLachlan R., Pozniak C. & Banniza S. Candidate effectors contribute to race differentiation and virulence of the lentil anthracnose pathogen *Colletotrichum lentis*. BMC Genom. 16, 628 (2015). 10.1186/s12864-015-1836-2 PMC4546252 26296655

[b12] Conesa A. . Blast2GO: a universal tool for annotation, visualization and analysis in functional genomics research. Bioinformatics 21 **(18)**, 3674–3676 (2005). 16081474 10.1093/bioinformatics/bti610

[b13] Syme R. A., Hane J. K., Friesen T. L. & Oliver R. P. Resequencing and comparative genomics of *Stagonospora nodorum*: sectional gene absence and effector discovery. G3 (Bethesda) 3, 959–969 (2013). 23589517 10.1534/g3.112.004994 PMC3689807

[b14] O’Connell R. . Lifestyle transitions in plant pathogenic *Colletotrichum* fungi deciphered by genome and transcriptome analyses. Nature Genet. 44, 1060–1065 (2012). 22885923 10.1038/ng.2372 PMC9754331

[b15] Perfect S. E., O’Connell R. J., Green E. F., Doering-Saad C. & Green J. R. Expression cloning of a fungal proline rich glycoprotein specific to the biotrophic interface formed in the Colletotrichum-bean interaction. Plant J. 15, 273–279 (1998). 9721685 10.1046/j.1365-313x.1998.00196.x

[b16] Stergiopoulos I. & de Wit P. J. G. M. Fungal effector proteins. Annu. Rev. Phytopathol. 47, 233–263 (2009). 19400631 10.1146/annurev.phyto.112408.132637

[b17] Koharudin L. M. . Structure-function analysis of a CVNH-LysM lectin expressed during plant infection by the rice blast fungus Magnaporthe oryzae. Structure 19, 662–674 (2011). 21565701 10.1016/j.str.2011.03.004 PMC3094573

[b18] Köller W. & Parker D. M. Purification and characterization of cutinase from Venturia inaequalis. Phytopathology 7, 278–283 (1989).

[b19] Teixeira P. J. P. L. The fungal pathogen *Moniliophthora perniciosa* has genes similar to plant *PR-1* that are highly expressed during its interaction with Cacao. PLoS One 7 **(9)**, e45929 (2012). 23029323 10.1371/journal.pone.0045929 PMC3447762

[b20] Breiteneder H. . The gene coding for the major birch pollen allergen Betv1, is highly homologous to a pea disease resistance response gene. EMBO J. 8 **(7)**, 1935–1938 (1989). 2571499 10.1002/j.1460-2075.1989.tb03597.x PMC401053

[b21] Allaire B. S. & Hadwiger L. A. Immunogold localization of a disease resistance response protein in *Pisum sativum* endocarp cells. Physiol. Mol. Plant Pathol. 44, 9–17 (1994).

[b22] de Jonge R. . Extensive chromosome reshuffling drives evolution of virulence in an asexual pathogen. Genome Res. 23, 1271–1282 (2013). 23685541 10.1101/gr.152660.112 PMC3730101

[b23] Orbach M. J., Farrall L., Sweigard J. A., Chumley F. G. & Valent B. A telomeric avirulence gene determines efficacy for the rice blast resistance gene *Pi-ta*. Plant Cell 12, 2019–2032 (2000). 11090206 10.1105/tpc.12.11.2019 PMC152363

[b24] Cuomo C. A. . The *Fusarium graminearum* genome reveals a link between localized polymorphism and pathogen specialization. Science 317, 1400–1402 (2007). 17823352 10.1126/science.1143708

[b25] Skamnioti P. . Genetics of avirulence genes in *Blumeria graminis* f. sp. *hordei* and physical mapping of *AVR*a22 and *AVR*a12. Fungal Genet. Biol. 45, 243–252 (2008). 18036855 10.1016/j.fgb.2007.09.011

[b26] Woloshuk C. P. & Kolattukudy P. E. Mechanism by which contact with plant cuticle triggers cutinase gene expression in the spores of *Fusarium solani* f. sp. pisi. Proc. Natl. Acad. Sci. USA 83, 1704–1708 (1986). 16593666 10.1073/pnas.83.6.1704 PMC323152

[b27] Wang C. L., Chin C. K. & Gianfagna T. Relationship between cutin monomers and tomato resistance to powdery mildew infection. Physiol. Mol. Plant Pathol. 57, 55–61 (2000).

[b28] Auyong A. S. M., Ford R. & Taylor P. W. J. The Role of Cutinase and its Impact on Pathogenicity of Colletotrichum truncatum. J. Plant Pathol. Microb. 6, 259 (2015).

[b29] Kwon S. J. GDSL lipase-like 1 regulates systemic resistance associated with ethylene signaling in Arabidopsis. Plant J. 58(2), 235–45 (2009). 19077166 10.1111/j.1365-313X.2008.03772.x

[b30] Rauscher M. . PR-1 protein inhibits the differentiation of rust infection hyphae in leaves of acquired resistant broad bean. Plant J. 19, 625–633 (1999). 10571848 10.1046/j.1365-313x.1999.00545.x

[b31] Niderman T. . Pathogenesis related PR-1 proteins are antifungal. Isolation and characterization of three 14-kilodalton proteins of tomato and of a basic PR-1 of tobacco with inhibitory activity against *Phytophthora infestans*. Plant Physiol. 108, 17–27 (1995). 7784503 10.1104/pp.108.1.17 PMC157301

[b32] Kiba A., Nishihara M., Nakatsuka T. & Yamamura S. Pathogenesis-related protein 1 homologue is an antifungal protein in Wasabia japonica leaves and confers resistance to Botrytis cinerea in transgenic tobacco. Plant Biotech. 24, 247–254 (2007).

[b33] Zhang Y., Goritschnig S., Dong X. & Li X. A gain-of-function mutation in a plant disease resistance gene leads to constitutive activation of downstream signal transduction pathways in suppressor of npr1-1, constitutive 1. Plant Cell 15, 2636–2646 (2003). 14576290 10.1105/tpc.015842 PMC280567

[b34] Swoboda I. . Bet v 1 proteins, the major birch pollen allergens and members of a family of conserved pathogenesis related proteins, show ribonuclease activity *in vitro*. Physiol. Plant 96, 433–438 (1996).

[b35] Bufe A., Spangfort M. D., Kahlert H., Schlaak M. & Becker W. M. The major birch pollen allergen, bet v 1, shows ribonuclease activity. Planta 199, 413–415 (1996). 8771801 10.1007/BF00195733

[b36] Hadwiger L. A. Anatomy of a nonhost disease resistance response of pea to Fusarium solani: PR gene elicitation via DNase, chitosan and chromatin alterations. Front Plant Sci. 6, 373 (2015). 26124762 10.3389/fpls.2015.00373 PMC4464173

[b37] Chang M. M., Chiang C. C., Martin M. W. & Hadwiger L. A. Expression of a pea disease resistance response protein in potatoes. Amer. Pot. J. 70, 635–647 (1993).

[b38] Liu J. J. & Ekramoddoullah A. K. M. The family 10 of plant pathogenesis-related proteins: Their structure, regulation, and function in response to biotic and abiotic stresses. Physiol. Mol. Plant Pathol. 68, 3–13 (2006).

[b39] Wilkins T. A. & Smart L. B. Isolation of RNA from plant tissue in *A laboratory guide to RNA:* Isolation, analysis, and synthesis (ed. Krieg P. A.) 21–41 (Wiley-Liss, 1996).

[b40] Hall T. A. BioEdit: a user-friendly biological sequence alignment editor and analysis program for Windows 95/98/NT. Nucleic Acids Symp. Ser. 41, 95–98 (1999).

[b41] Min X. J., Butler G., Storms R. & Tsang A. OrfPredictor: predicting protein-coding regions in EST-derived sequences. Nucl. Acids Res. 33, W677–80 (2005). 15980561 10.1093/nar/gki394 PMC1160155

[b42] Camacho C. . BLAST+: architecture and applications. BMC Bioinform. 10, 421 (2009). 10.1186/1471-2105-10-421 PMC2803857 20003500

[b43] Kaneshisa M. & Goto S. KEGG: Kyoto Encyclopedia of Genes and Genome. Nucl. Acids Res. 28, 27–30 (2000). 10592173 10.1093/nar/28.1.27 PMC102409

[b44] Kaneshisa M., Goto S., Sato Y., Furumichi M. & Tanabe M. KEGG for integration and interpretation of large scale molecular datasets. Nucl. Acids Res. 40, D109–14 (2012). 22080510 10.1093/nar/gkr988 PMC3245020

[b45] Hunter S. . InterPro: the integrative protein signature database. Nucl. Acids Res. 37 (suppl 1), D211–D215 (2009). 18940856 10.1093/nar/gkn785 PMC2686546

[b46] Horton P. . WoLF PSORT: protein localization predictor. Nucleic Acids Res. 35, W585–W587 (2007). 17517783 10.1093/nar/gkm259 PMC1933216

[b47] Li H. . 1000 Genome Project Data Processing Subgroup: The sequence alignment/Map format and SAMtools. Bioinformatics 25, 2078–2079 (2009). 19505943 10.1093/bioinformatics/btp352 PMC2723002

[b48] Livak K. J. & Schmittgen T. D. Analysis of relative gene expression data using real-time quantitative PCR and the 2^−ΔΔCT^ method. Methods 25, 402–8 (2001). 11846609 10.1006/meth.2001.1262

[b49] Stamatakis A. RAxML-VI-HPC: maximum likelihood-based phylogenetic analyses with thousands of taxa and mixed models. Bioinformatics 22(21), 2688–2690 (2006). 16928733 10.1093/bioinformatics/btl446

[b50] Roy A., Kucukural A. & Zhang Y. I-TASSER: a unified platform for automated protein structure and function prediction. Nat. Protoc. 5, 725–738 (2010). 20360767 10.1038/nprot.2010.5 PMC2849174

