# Supplementary material for: Transcriptome analysis reveals a complex interplay between resistance and effector genes during the compatible lentil-Colletotrichum lentis interaction
Source: Sci Rep. 2017 Feb 10;7:42338. doi: 10.1038/srep42338 (PMC5301223; doi:10.1038/srep42338)
Supplement: Supplementary Information [file srep42338-s1.pdf]

Transcriptome analysis reveals a complex interplay between resistance and effector genes during the compatible lentil-*Colletotrichum lentis* interaction

Vijai Bhadauria[1,2], Perumal Vijayan[1], Yangdou Wei[3] & Sabine Banniza[1]*

[1]Crop Development Centre/Department of Plant Sciences, University of Saskatchewan, Saskatoon, Canada

[2]Current address: Semiarid Prairie Agricultural Research Centre, Agriculture and Agri-Food Canada, Swift Current, Canada

[3]Department of Biology, University of Saskatchewan, Saskatoon, Canada

*Correspondence and requests for materials should be addressed to SB (email: sabine.banniza@usask.ca)

Suppl. Table 1. Detailed gene ontology (level 2) term descriptions of *Colletotrichum lentis* (isolate CT-30 / race 0) unigenes.

GO-id	GO-term (Biological processes)	Seqs
GO:0008152	metabolic process	712
GO:0009987	cellular process	609
GO:0044699	single-organism process	539
GO:0071840	cellular component organization or biogenesis	228
GO:0051179	localization	208
GO:0065007	biological regulation	170
GO:0050896	response to stimulus	101
GO:0032502	developmental process	44
GO:0023052	signaling	36
GO:0000003	reproduction	29
GO:0032501	multicellular organismal process	28
GO:0051704	multi-organism process	21
GO:0022414	reproductive process	17
GO:0040007	growth	11
GO:0022610	biological adhesion	1
GO:0040011	locomotion	1
**GO-id**	**GO-term (Molecular functions)**	**Seqs**
GO:0003824	catalytic activity	536
GO:0005488	binding	422
GO:0005215	transporter activity	86
GO:0005198	structural molecule activity	60
GO:0001071	nucleic acid binding transcription factor activity	23
GO:0098772	molecular function regulator	18
GO:0009055	electron carrier activity	6
GO:0016209	antioxidant activity	6
GO:0060089	molecular transducer activity	5
GO:0000988	protein binding transcription factor activity	5
GO:0031386	protein tag	4
GO:0045182	translation regulator activity	1
**GO-id**	**GO-term (Cellular components)**	**Seqs**
GO:0005623	cell	562
GO:0043226	organelle	483
GO:0032991	macromolecular complex	280
GO:0016020	membrane	236
GO:0031974	membrane-enclosed lumen	94
GO:0005576	extracellular region	17
GO:0009295	nucleoid	8
GO:0019012	virion	6

GO:0044215	other organism	3

Suppl. Table 2. Detailed gene ontology (level 2) term descriptions of *Lens culinaris* cultivar Eston unigenes.

GO-id	GO-term (Biological processes)	Seqs
GO:0008152	metabolic process	450
GO:0009987	cellular process	342
GO:0044699	single-organism process	277
GO:0050896	response to stimulus	151
GO:0065007	biological regulation	109
GO:0051179	localization	100
GO:0071840	cellular component organization or biogenesis	61
GO:0023052	signaling	33
GO:0032502	developmental process	31
GO:0051704	multi-organism process	27
GO:0032501	multicellular organismal process	26
GO:0022414	reproductive process	17
GO:0002376	immune system process	16
GO:0040007	growth	11
GO:0040011	locomotion	3
GO:0000003	reproduction	1
**GO-id**	**GO-term (Molecular functions)**	**Seqs**
GO:0003824	catalytic activity	330
GO:0005488	binding	322
GO:0005215	transporter activity	35
GO:0005198	structural molecule activity	28
GO:0016209	antioxidant activity	14
GO:0001071	nucleic acid binding transcription factor activity	12
GO:0098772	molecular function regulator	9
GO:0060089	molecular transducer activity	6
GO:0009055	electron carrier activity	6
GO:0045735	nutrient reservoir activity	2
GO:0000988	protein binding transcription factor activity	1
**GO-id**	**GO-term (Cellular components)**	**Seqs**
GO:0005623	cell	265
GO:0043226	organelle	162
GO:0016020	membrane	117
GO:0032991	macromolecular complex	69
GO:0005576	extracellular region	34
GO:0031974	membrane-enclosed lumen	19
GO:0055044	symplast	15
GO:0030054	cell junction	15
GO:0031012	extracellular matrix	2

GO:0019012	virion	2

Suppl. Table 3. Primers used in the study.

Primer	Sequence (5'-3')
ClCE0-5_F	TGCGGACTCAGCCTACAATC
ClCE0-5_R	TACGGTGTTTCCGACCGATG
ClCE0-8_F	TTAACGCCTGTGGCCTGTAG
ClCE0-8_R	ATGCATTGTCGAGCTGTCCA
ClCE0-13_F	GCCACTACACCCAGATCGTC
ClCE0-13_R	TTCTCGCCGATGAAGTTGCC
ClCE0-15_F	ATCGGAATCGAATCGGCACA
ClCE0-15_R	TAGTGGAAGAGGGTGGCGTA
LcSnc1_F	TTGCAGATGCCATGCTTGTT
LcSnc1_R	GTTAAAGGAGGACCCACCTCA
LcDirigent_F	TCCACTTTGGGGACTTGGTAG
LcDirigent_R	GATCAGCGCCAGCAAAAGTAA

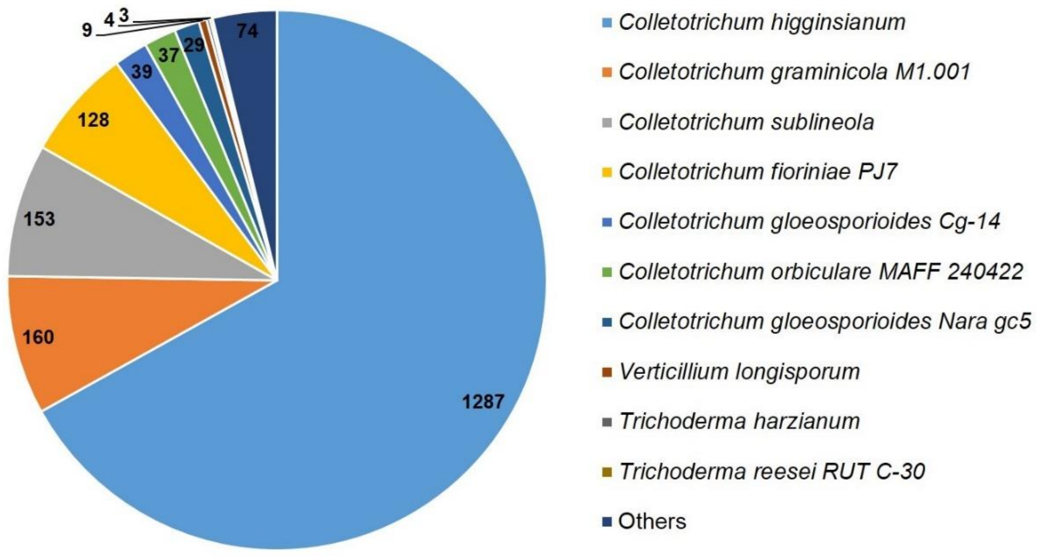

Suppl. Fig. 1. The top ten fungal species in blast hits classified based on species resulting from the query of *Colletotrichum lentis* unigenes against the non-redundant protein database using blastx. The number within or next to slices represent the number of unigenes.

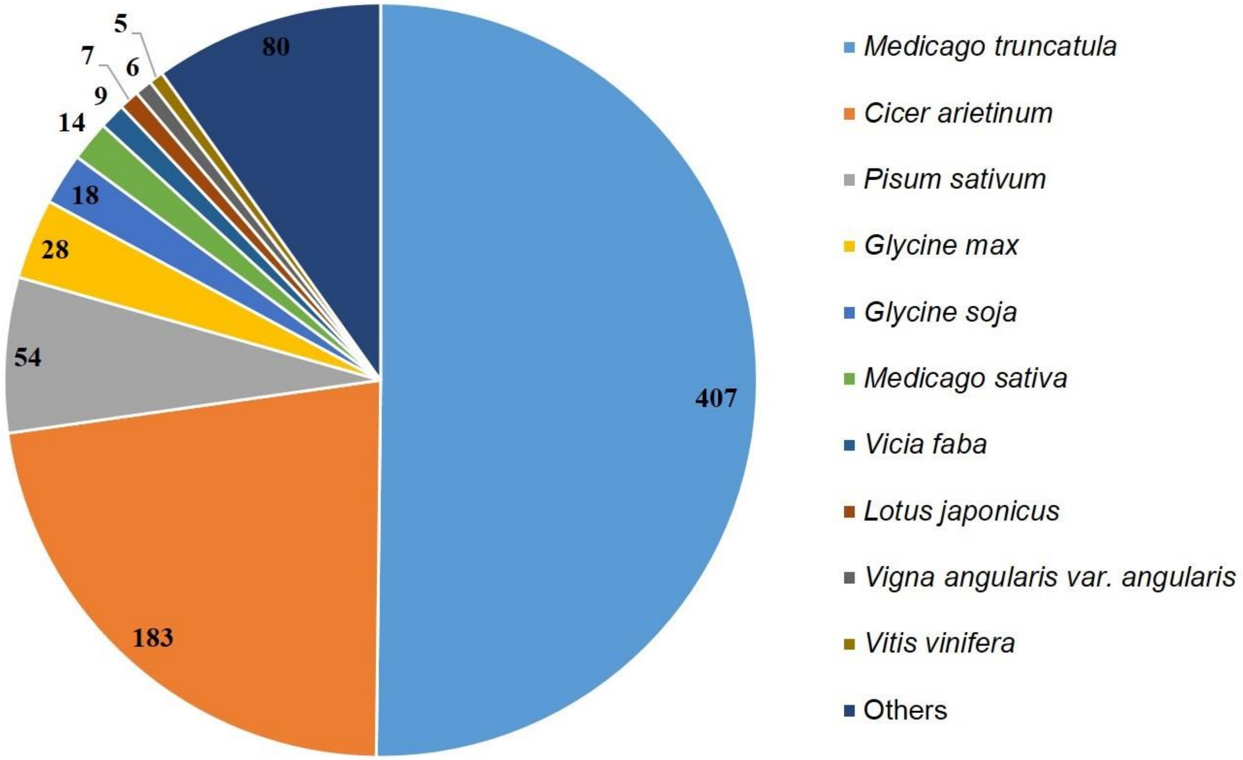

Suppl. Fig. 2. The top ten plant species in blast hits classified based on the species resulting from the query of *Lens culinaris* cultivar Eston unigenes against the non-redundant protein database using blastx. The number within or next to slices represent the number of unigenes.

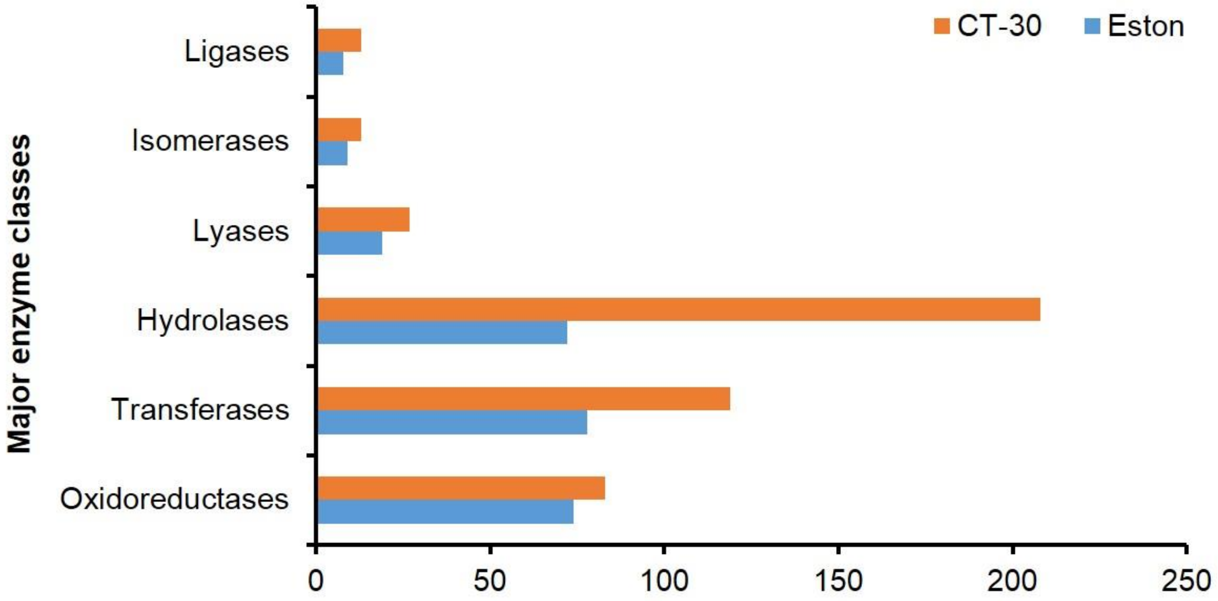

Suppl. Fig. 3. Comparative analysis of major enzyme classes. Proteins encoded by *Colletotrichum lentis* and *Lens culinaris* unigenes were mapped onto the KEGG pathways, and major enzyme classes were compiled as a comparative bar chart.

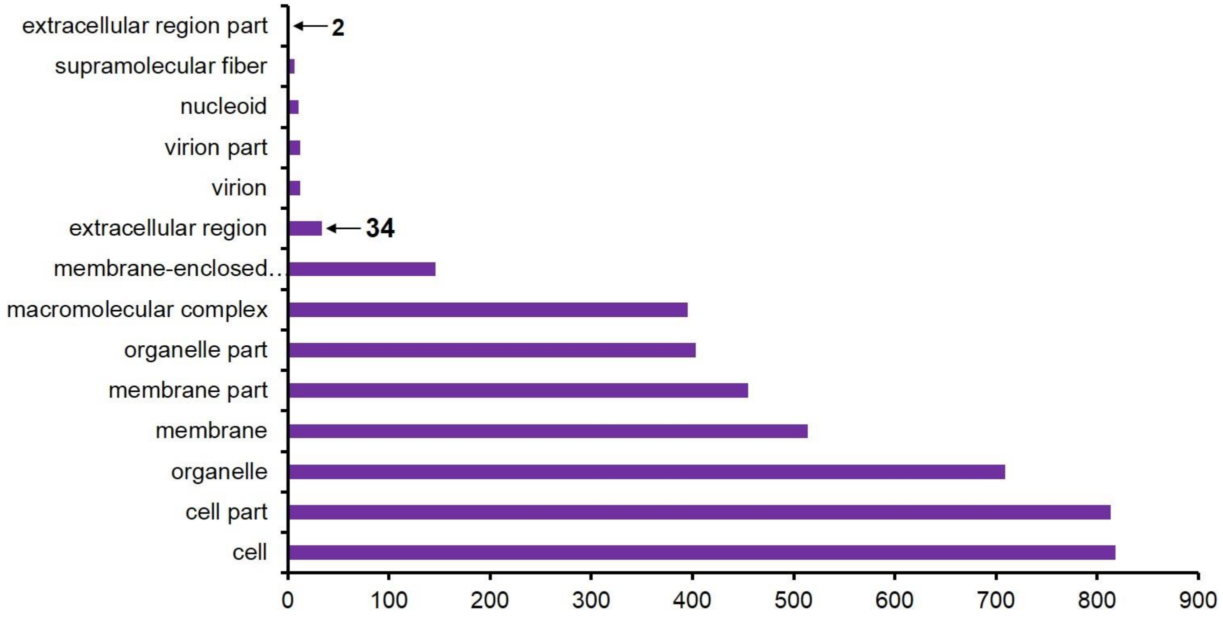

Suppl. Fig. 4. Cellular components derived from level 2 gene ontology (GO) descriptions. Proteins encoded by *Colletotrichum lentis* unigenes were classified based on their subcellular localization using the blast2go software.

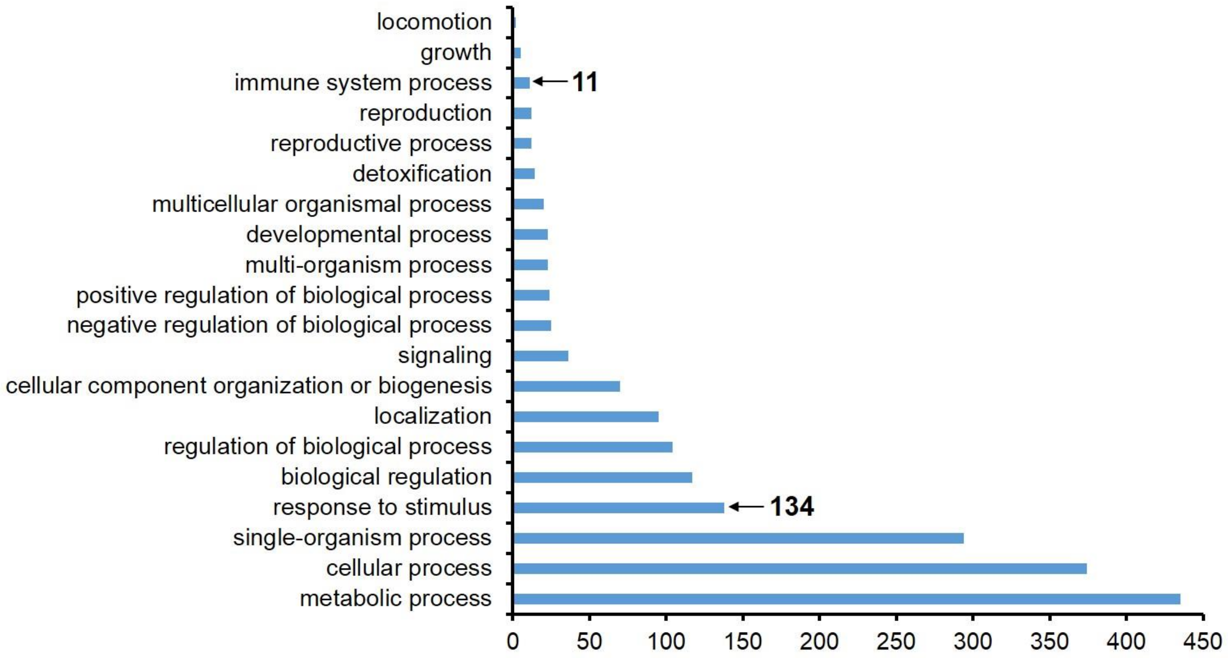

Suppl. Fig. 5. Biological processes derived from level 2 gene ontology descriptions. Proteins encoded by *Lens culinaris* cultivar Eston unigenes were classified based on their implication in various biological processes using the blast2go software.

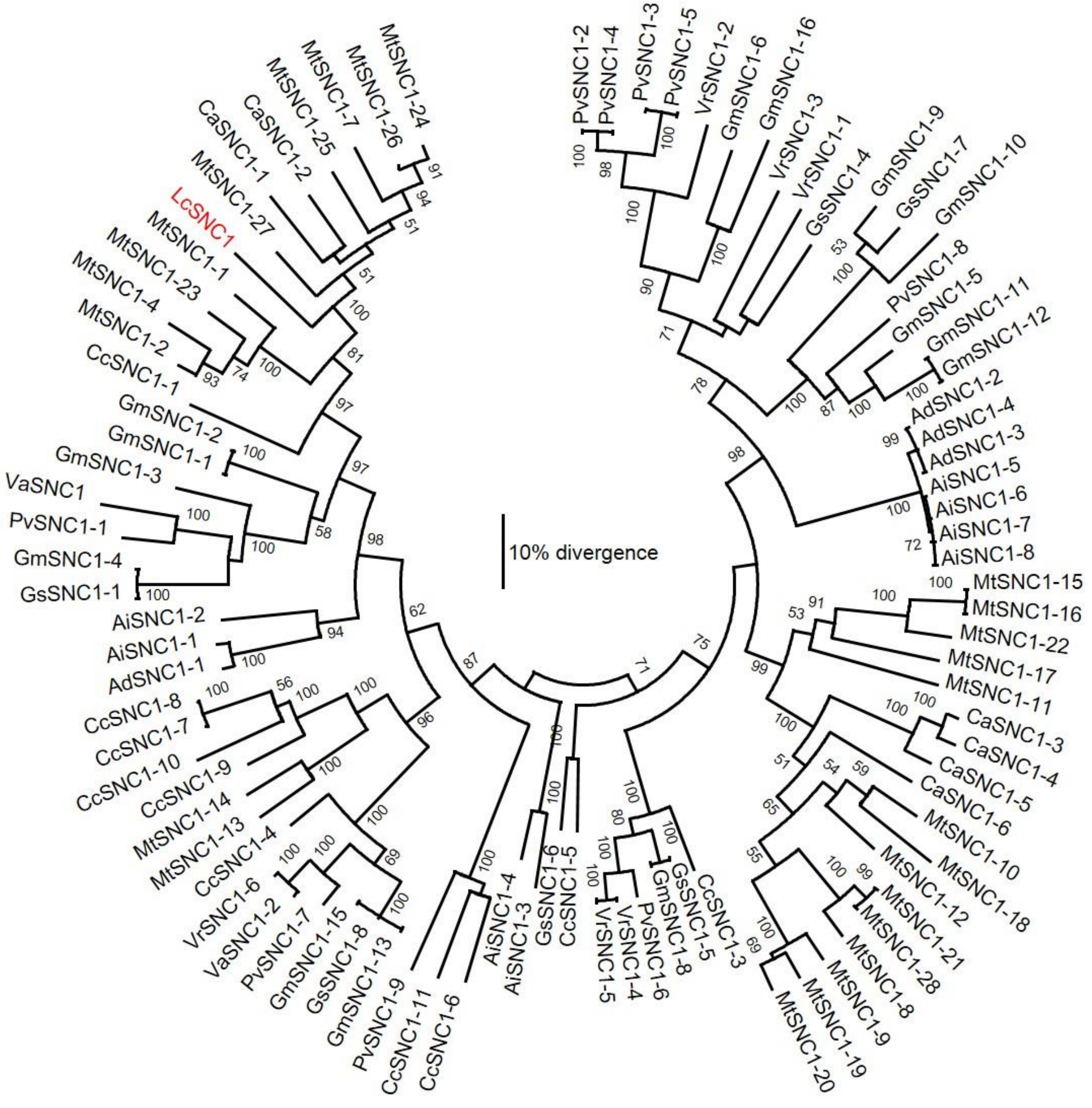

Suppl. Fig. 6. Phylogenetic analysis of homologs of LcSNC1 from *Lens culinaris*. An unrooted phylogenetic tree was constructed using the randomized axelerated *maximum likelihood (RAxML) from LcSNC1 homologs derived from the non-redundant protein data available at the NCBI using blastp with an e value of 1e-30*. The bar indicates mean sequence divergence. The SNC1 protein is prefixed with the species name: Ps, *Pisum sativum*; Pv, *Phaseolus vulgaris*; Ca, *Cicer arietinum*, Gm, *Glycine max*; Gs, *Glycine soja*; Mt, *Medicago truncatulata*; Cc, *Cajanus cajan*; Ad, *Arachis duranensis*; Ai, *A. ipaensis*; Vr, *Vigna radiate*; and Va, *V. angularis*.

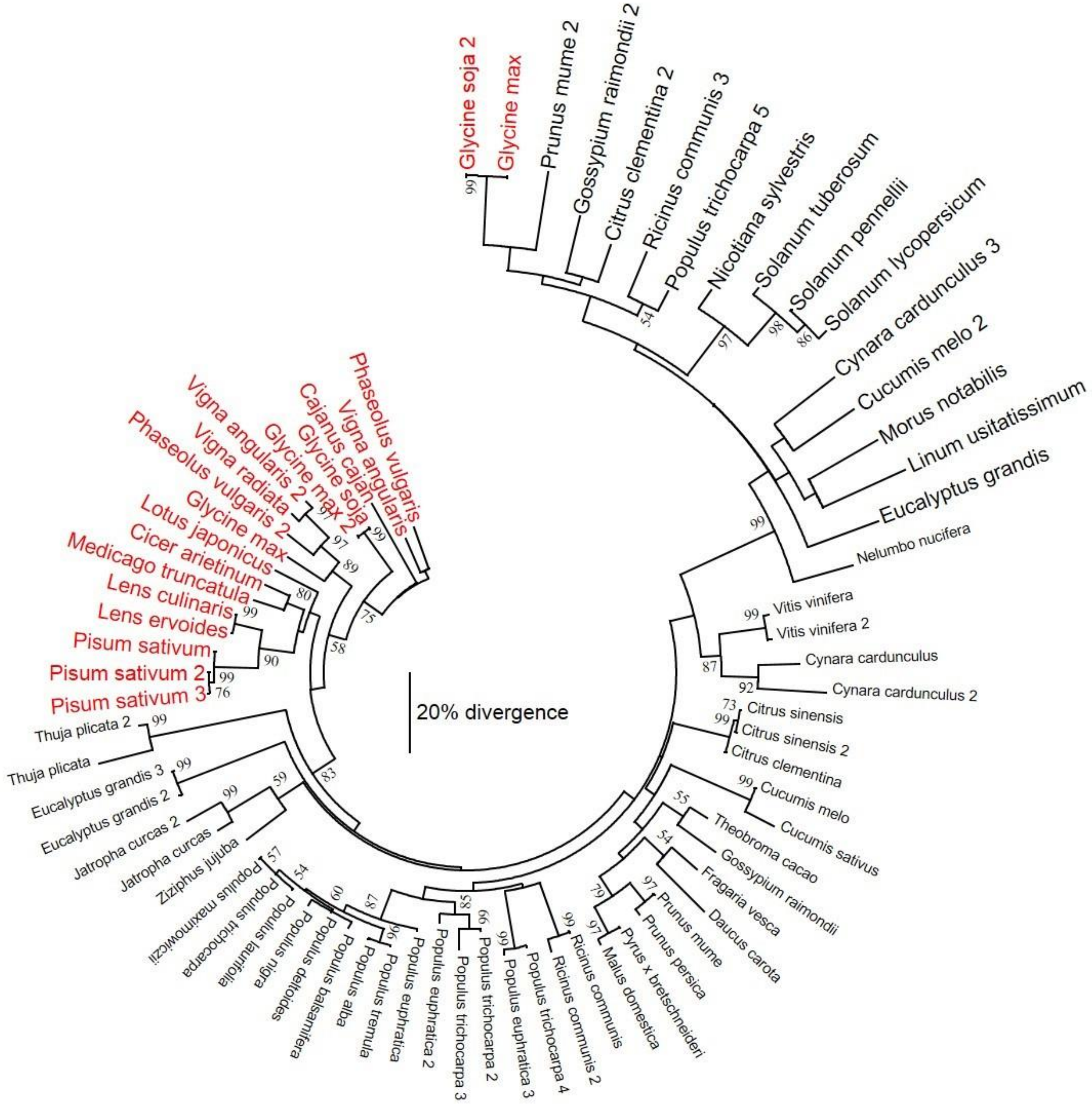

Suppl. Fig. 7. Phylogenetic analysis of homologs of LcDirigent from *Lens culinaris*. An unrooted phylogenetic tree was generated using the randomized axelerated *maximum likelihood (RAxML) from LcDirigent homologs derived from the non-redundant protein data available at the NCBI using blastp with an e value 1e-30*. The bar indicates mean sequence divergence.
